# Interactions at the Streptomyces – animal interface: ecology, defence and disease

**DOI:** 10.1099/mic.0.001747

**Published:** 2026-08-07

**Authors:** Ainsley D.M. Beaton, James T. Croxford, Alejandra C. Díaz de Aguinaga, Emily Horsburgh, David R. Mark, Lia S. McQueary, Kaisha R. Murray-Clelland, Samantha K. Tucker, Andrew J. Roe, Rebecca E. McHugh

**Affiliations:** 1 School of Infection and Immunity, University of Glasgow, Glasgow, G12 8TA, UK; 2 James Watt School of Engineering, University of Glasgow, Glasgow, G12 8QQ, UK

**Keywords:** animal microbiomes, chemical ecology, *Streptomyces*

## Abstract

*Streptomyces* are filamentous, spore-forming members of the *Actinomycetota*, renowned for their capacity to produce chemically diverse, specialized metabolites with medically important properties. Traditionally, *Streptomyces* have been viewed as soil-dwelling microbes, and their roles in soil ecology, plant health and plant disease have been extensively studied. However, advances in metagenomic sequencing and molecular approaches have greatly expanded our ability to investigate interkingdom interactions between *Streptomyces* and more complex organisms, including animals. In recent years, a growing body of work has revealed diverse and often intimate associations between *Streptomyces* and members of the Animalia. These include interactions with microfauna such as nematodes (*Nematoda*), insects (*Insecta*), including bees and ants, mammals such as bats (*Chiroptera*) and humans (*Homo sapiens*). This review consolidates our current knowledge of *Streptomyces* – animal interactions, with a particular focus on chemical ecology and the roles of specialized metabolites in shaping these relationships. This work highlights the emerging body of work investigating the role of *Streptomyces* ecology beyond soil ecosystems and draws attention to the importance of exploring non-traditional niches, including animal-associated microbiomes, to deepen our understanding of microbial–animal interactions and to expand opportunities for natural product discovery.

## Graphical Abstract

Phylogenetic tree highlighting members of the kingdom Animalia with characterized *Streptomyces* interactions. Images have been coloured using stereoimages of *Streptomyces* colonies.

## Introduction

Interactions between bacteria and animals are ancient and have profoundly shaped life on Earth. These relationships can range from pathogenic to mutualistic, with costs and benefits for both animal hosts and their associated micro-organisms. Advances in metagenomic and molecular analyses have expanded our understanding of these interactions and have facilitated studies which characterize previously overlooked constituents of the microbiota [1]. Research on *Streptomyces* has traditionally focused on both their complex life cycle – transitioning between multicellular vegetative hyphae into unicellular and metabolically quiescent spores – and their prodigious capacity for biosynthesis of many complex antibiotic molecules. Beyond soil, however, they have also been associated with a wide range of animal-associated ecological niches [2–6]. Historically, owing to their capacity for sporulation, and thus capability to survive in harsh environments, these bacteria were often dismissed as contaminants or transient ‘passers-through’; however, accumulating evidence indicates that *Streptomyces* are true, ecologically relevant members of animal-associated microbial communities. The following sections examine how these associations manifest across different animal systems, emphasizing the chemical and ecological mechanisms that underpin five of the best-characterized *Streptomyces*–animal interactions.

## Interactions between *Streptomyces* and nematodes (*Nematoda*)

The phylum *Nematoda* constitutes a dominant lineage of invertebrates, occurring throughout nearly all terrestrial and aquatic ecosystems [7]. Commonly known as roundworms, nematodes constitute a central component of soil microfauna, which are microscopic animals that play key roles in ecosystem function. Their ecological success stems largely from their versatile feeding strategies, which allow them to gain an ecological niche within a wide range of habitats. Accordingly, nematodes are often classified by their nutritional sources. Microbivorous nematodes feed on environmental micro-organisms, including bacteria (bacterivores) and fungi (fungivores), whereas predatory nematodes consume other nematodes and soil-dwelling organisms. Parasitic nematodes, perhaps the most widely studied group, depend on plant (phytonematodes) or animal hosts for survival [7]. In humans, lymphatic filariasis represents an example of parasitic nematodes. In its chronic stage, the disease can lead to elephantiasis, the most widely recognized clinical manifestation [8]. Transmission occurs through mosquito vectors, and ~90% of cases are attributed to the filarial worm *Wuchereria bancrofti* [8]. In plants, *Pratylenchus coffeae* is a plant-parasitic nematode (PNN) associated with yield reduction and significant economic losses in coffee production. This parasite feeds directly on root tissues inducing extensive root system damage, impairing water and nutrient uptake [9].

Whilst evidence for direct colonization of nematodes by *Streptomyces* is limited, many natural products derived from *Streptomyces* are reported to exhibit nematocidal and nemato-toxic activity [10]. One such example is avermectin, produced by *Streptomyces avermitilis*. This compound was isolated in 1975 by Ōmura *et al*., representing a historic milestone that led to the synthesis of its chemical derivative, ivermectin [11]. This drug exhibited reduced toxicity in humans and greater specificity against parasitic infections. A notable example is onchocerciasis (river blindness), for which diethylcarbamazine had previously been the treatment of choice. However, this drug was associated with severe adverse effects including allergic reactions and potentially fatal complications due to the massive die-off of microfilariae [11]. This development demonstrates the pivotal role of *Streptomyces*-derived compounds in advancing antiparasitic therapy. Furthermore, ivermectin has been successfully employed in mass drug administration programmes, significantly reducing the global burden of neglected tropical diseases [12, 13]. However, there are concerns regarding toxicity and the environmental impact of these compounds on biodiversity, particularly their effects on non-target organisms such as beneficial soil invertebrates and aquatic ecosystems [14]. Thus, as the study of alternative methods for the control of agricultural pests emerges, there is growing interest in developing more targeted and environmentally sustainable biocontrol strategies that harness the natural antagonistic properties of *Streptomyces* while minimizing ecological disruption [15].

*Meloidogyne enterolobii* is among the most virulent root-knot nematodes [16]. This nematode is known to cause significant plant health decline and reduced growth, as well as an increased risk of secondary infection. Guava crops benefit from the relationship between *Streptomyces* and *M. enterolobii*, as *Streptomyces* have been reported to suppress and prevent nematode infestation in the plant. Research conducted by Mani *et al*. [10] found that *Streptomyces* strains, such as *Streptomyces rochei* (KM2) isolated from the rhizosphere of guava plants, suppressed *M. enterolobii* infection and exhibited inhibition of egg hatching and nematocidal activity against juvenile populations of *M. enterolobii* [15, 17]. Moreover, these strains produced secondary metabolites promoting plant growth and resilience against nematode infection. Similar interactions are also observed in maize plants resisting *M. incognita* infection [18]. In this instance, *Streptomyces* were shown to express secondary metabolites with nematocidal activity and exhibit nematode-suppressing activity which reduced root gall formation by 58.7%. Furthermore, these *Streptomyces* strains were shown to promote plant immunity via metabolites targeting the jasmonic acid signalling pathway.

*Streptomyces* have also been found in the cyst microbiome of soybean cyst nematodes (*Heterodera glycines*), an agricultural pest targeting soya crops [19]. Moreover, research by Trejo-Meléndez *et al*. (2025) [19] found that *Streptomyces* in combination with other rhizosphere bacteria were capable of controlling *H. glycines* infection [19]. This evidence reinforces the view of ‘old enemies’ between *Streptomyces* and nematodes and how this antagonistic relationship is of great relevance in the agricultural field against pests.

Bacterivorous nematodes (BNs) primarily feed on bacteria in their environment. These nematodes play a fundamental role in soil function, contributing to nutrient cycling and enhancing soil-available nitrogen and phosphorus. They can also act as carbon sequestration systems within the soil and are key regulators of bacterial communities, increasing bacterial biomass [20]. In agricultural microcosm studies, the presence of the BN *Acrobeloides buetschlii* results in significant microbial community structure reshape as a result of grazing on highly abundant taxa [21]. In contrast, the composition of *Actinomycetota*, including *Streptomyces*, and other dominant Gram-positive taxa were not affected by *A. buetschlii* [21]. These findings are likely attributed to the selective feeding behaviour of these roundworms, which avoid Gram-positive bacteria and instead prey on Gram-negative bacteria [20, 21]. Moreover, these nematodes also exhibit a preference for unicellular bacteria and seemingly avoid filamentous bacteria, such as *Streptomyces*. Furthermore, the biosynthetic activity of *Streptomyces* is an active defence against nematode predation. The bacterivorous nematode *Caenorhabditis elegans* can sense and dodecanoic acid produced by streptomycetes and consequently elicits an avoidance response via the chemosensory receptor SRB-6 [18, 22].

This *Streptomyces*-avoidance system in *C. elegans* might be the result of an evolutionary strategy to prevent damage from toxic *Streptomyces*. Alternatively, the production of these molecules could reflect *Streptomyces* adaptation of its biosynthetic capacity to deter predation. In previous research by Caldwell *et al*. [23], both *Streptomyces venezuelae* and its secreted metabolites exerted neurotoxic effects in *C. elegans*, resulting in dopaminergic neuron degeneration [23]. Due to concerns in the increased prevalence of Parkinson’s disease in rural communities, Watkins *et al*. [24] investigated the potential effect that *Streptomyces* metabolites have on dopaminergic neuron damage using *C. elegans* as a model organism [24]. It was observed that 29.3% of the taxonomically unique *Streptomyces* spp. isolated from the agricultural, natural and urban soil samples caused neurodegeneration in *C. elegans*. Moreover, urban soils harboured a lower proportion of neurodegenerative-metabolite-producing *Streptomyces* strains than agricultural and natural soils. The authors of this study hypothesize that the presence of agricultural chemicals (pesticides, fertilizers) may increase selective pressure for the carriage of neurodegenerative compounds. These observations highlight genome-level variation in *Streptomyces*, reflecting on distinct ecological pressures.

Collectively, these studies highlight that *Streptomyces* interact extensively with nematodes and act as a source of nematode-inhibitory natural products, a biocontrol agent against PNNs and a protective symbiont that enhances host resistance to nematode predation. Through the production of diverse secondary metabolites, beyond antagonism, emerging evidence suggests that *Streptomyces*–nematode relationships are context-dependent, ranging from defensive mutualism to chemical warfare, and are likely influenced by environmental conditions and community composition. At present, there is little evidence that relationships with *Streptomyces* confer benefits to nematodes themselves, other than providing a food source. These studies also suggest that *Streptomyces* may utilize secondary metabolites as virulence factors within these systems. Therefore, future studies of these interactions could provide practical and sustainable solutions to nematode-mediated diseases of animals and plants.

## Interactions between *Streptomyces* and *Formicidae* (ants)

Complex agricultural and medical systems observed in ant colonies represent evolutionary innovations that predate the emergence of analogous human infrastructures by millions of years. Ants of the tribe Attini have cultivated fungi as a primary food source for their larvae for millions of years and, in parallel, have harnessed *Actinomycetota* to produce antifungal compounds that protect their fungal gardens from pathogenic invasion [25].

The Attini comprise nearly 50 extant genera, including the leaf-cutting ants *Acromyrmex* and *Atta*, and are thought to have evolved fungiculture ~66 million years ago. These ants cultivate fungal symbionts, most commonly *Leucoagaricus gongylophorus*, which produce nutritionally rich gongylidia that serve as the primary nutritional source for the colony [25]. Since these fungal monoculture gardens are vulnerable to specialized pathogens, often fungi of the genus *Escovopsis*, fungus-growing ants have evolved multilayered defensive strategies that include symbiotic microbes.

Early work by [26] established the paradigm of microbial defence in leaf-cutting ants, demonstrating that the characteristic ‘waxy bloom’ on the cuticle of *Acromyrmex* ants consists of filamentous *Actinomycetota*, initially described as *Streptomyces* and later reclassified as *Pseudonocardia* [27]. These bacteria are vertically transmitted by queens, selectively inhibit *Escovopsis* and protect the fungal cultivar, reframing ant fungiculture as a tripartite symbiosis involving ants, fungi and defensive bacteria.

Subsequent studies suggested that this relationship also truly includes *Streptomyces* as an additional and potentially more dynamic, defensive partner. Kost *et al*. [28] showed that *Streptomyces* isolated from the cuticle of *Acromyrmex octospinosus* frequently inhibited *Escovopsis*, including strains recovered from both attine and non-attine ants [28]. Similar findings were reported from *Trachymyrmex* nests, where *Streptomyces* represented approximately two-thirds of isolated *Actinomycetota* [29]. D’Angelo *et al*. (2016) [30] further demonstrated that *Streptomyces* isolated directly from the cuticle of multiple *Acromyrmex* species inhibited not only *Escovopsis* but also a broader range of filamentous fungi carried by foraging ants, including *Aspergillus* and *Penicillium*, supporting a role for *Streptomyces* in generalized antifungal defence [30].

The functional importance of *Streptomyces*-derived metabolites in these systems is underscored by chemical characterization studies. Haeder *et al*. [31] identified candicidin macrolides as the basis of anti-*Escovopsis* activity in *Streptomyces* sp. Ao10, and candicidin-producing strains were later recovered from geographically distinct *A. octospinosus* populations [31, 32]. However, variability exists: *Streptomyces* isolates from *Acromyrmex lundii* did not inhibit *Escovopsis* [33], and follow-up work showed that neither candicidin nor antimycin was responsible for antifungal activity in certain strains, suggesting additional, unidentified metabolites [34].

Large-scale surveys reinforce the prevalence of *Streptomyces* in ant-associated microbiomes. [35] recovered over 800 *Actinomycetota* isolates from three ant–plant–fungus mutualisms across South America and Africa, with *Streptomyces* dominating across ant species, life stages and geographic regions [35]. While many isolates showed antifungal activity, others displayed cellulose-degrading capacity, indicating roles in both defence and nutrient processing. Mining of previously isolated ant-derived *Streptomyces* strains has further revealed extensive cryptic biosynthetic potential, uncovering compounds such as lobophorins, chromomycins, benzanthrins, cervimycins, vicenistatin, warkmycins and sipanmycins A and B [36, 37].

Although antifungal activity dominates these systems, *Streptomyces* from leaf-cutting ants are not restricted to targeting eukaryotes. Schoenian et al. (2010) demonstrated that *Streptomyces* isolated from *Acromyrmex* ants produce antibacterial compounds, including actinomycins and valinomycin, which inhibit soil bacteria such as *Bacillus subtilis* and competing Actinomycota, alongside antimycins active against *Escovopsis weberi* [38]. Insecticidal activity has also been reported: *Streptomyces caniferus*, isolated from *Acromyrmex coronatus*, produces photoactive porphyrins that cause high mortality in *Spodoptera frugiperda* larvae via photodynamic generation of reactive oxygen species [39]. A summary of compounds identified from ant-associated *Streptomyces* is given in Table 1.

**Table 1.** Summary of compounds produced by ant-associated *Streptomyces* and their activity

Ant species/system	*Streptomyces* species/strain	Compound produced	Compound type/activity	Reference
*A. octospinosus*	*Streptomyces* sp. Ao10	Candicidin	Antifungal (anti-Escovopsis)	Haeder *et al*., 2009 [31]
*A. octospinosus*	*Streptomyces* spp.	Candicidin, antimycins	Antifungal	Barke *et al*., 2010; [32] Siepke *et al*., 2022
*Acromyrmex* spp.	Multiple *Streptomyce*s isolates	Actinomycins, valinomycin, antimycins	Antibacterial; antifungal	Schoenian *et al*., 2010 [38]
*A. coronatus*	*S. caniferus*	Porphyrins (e.g. coproporphyrins)	Insecticidal (photoactivated)	[39]
*Attini ants* (multiple spp.)	*Streptomyces* sp. CS113	Diperamycin	Antibiotic	García-Gutiérrez *et al*., 2024
*Attini ants*	Multiple *Streptomyces* spp.	Lobophorins, chromomycins, benzanthrins, cervimycins, vicenistatin, warkmycins, sipanmycin A, B	Antibiotic; antifungal	Malmierca *et al*., 2018 [36]
*Camponotus japonicus*	*Streptomyces amphotericinicus*	Amphotericin	Antifungal (clinical polyene)	Cao *et al*., 2017
*Camponotus japonicus*	*Streptomyces capitiformicae*	Angucyclinones	Antibiotic	Jiang *et al*., 2018
*Camponotus vagus*	*Streptomyces* sp.	Nybomycin	Antibiotic; cytotoxic	Zakalyukina *et al*., 2019
*Camponotus vagus*	*Streptomyces albidoflavus* A10	Antimycins	Antifungal	Baranova *et al*., 2020
*Camponotus kiusiuensis* (gut)	*Streptomyces* sp. STA1	Camporidines A, B	Antimetastatic; anti-inflammatory	Hong *et al*., 2019 [107]
*Tetraponera penzigi* (plant ant)	*Streptomyces formicae* KY5	Formicamycins; formicapyridines	Antibiotic (MRSA, VRE)	[47, 48]
*F. yessensis* (gut)	*Streptomyces* sp. BA01	Formicolides A, B	Antioxidative; anti-angiogenic	An *et al*., 2020
*F. yessensis* (gut)	*Streptomyces* sp.	Formicins A–C	Anticancer	[52]
*Lasius fuliginosus*	*Streptomyces lasiicapitis*	Kanchanamycin	Antibiotic	Ye *et al*., 2017 [108]
*Lasius niger* (nest)	*Streptomyces antibioticus-*like	Actinomycins	Antibiotic	[50]

MRSA, methicillin-resistant *S. aureus*; VRS, vancomycin-resistant *Enterococcus*.

Collectively, these studies reveal fungus-growing ant nests as chemically dynamic environments shaped by complex, evolving multi-species interactions. Defensive *Actinomycetota*, including *Pseudonocardia* and *Streptomyces*, produce antifungal polyenes, depsipeptides and other antibiotics [40–42]. Imaging MS has demonstrated that these metabolites co-localize at microbial interaction zones on the ant cuticle, providing direct evidence of *in situ* chemical warfare rather than artefacts of laboratory assays.

Although *Attini* ants dominate the literature, *Streptomyces*–ant associations extend well beyond fungiculture. Fungus-growing Allomerus ants, which cultivate Chaetothyriales fungi to construct ambush traps, harbour cuticle-associated *Streptomyces* and *Amycolatopsis* that inhibit non-cultivar fungi [34]. Scavenger ants (*Paratrechina longicornis*) preferentially harbour *Streptomyces* relative to surrounding soil communities, suggesting selective recruitment [43]. *Streptomyces* have also been identified in association with *Trachymyrmex septentrionalis* [44].

Perhaps the most clinically promising ant-derived discovery is the formicamycins, produced by *Streptomyces formicae* isolated from the African plant-ant *Tetraponera penzigi*. These halogenated aromatic polyketides show potent activity against methicillin-resistant *Staphylococcus aureus* and vancomycin-resistant *Enterococcus*, exhibit low resistance potential and have been the subject of extensive biosynthetic, regulatory and engineering studies, establishing this system as a leading example of translational potential from ant-associated *Streptomyces* [45–49].

Additional ant systems, including *Lasius niger*, *Formica yessensis* and *Polyrhachis lamellidens*, continue to reveal *Streptomyces* with broad phylogenetic diversity and metabolic capacity, producing actinomycins, formicolides, colibrimycin and pipericines, among others [50–52].

Overall, the evidence strongly suggests that *Streptomyces* are not recruited by ants as fixed, vertically transmitted symbionts, but rather as functionally selected, environmentally acquired partners. Their repeated recovery across ant taxa, body sites and geographic regions, combined with extraordinary biosynthetic diversity and amenability to pathway mining, positions ant-associated *Streptomyces* as a mobile, chemically driven system and a rich source of novel antimicrobial scaffolds. Fungus-farming ants provide well-known examples of host-associated *Streptomyces* producing defensive antimicrobial compounds; however, it remains unclear whether these strains are genuinely enriched in bioactive compounds and BGCs relative to free-living isolates, or whether this pattern partly reflects targeted sampling of chemically active symbionts.

## *Streptomyces* associations with family Apidae (bees)

Unlike ants, which are exclusively eusocial, colonial insects, bees and wasps represent hymenopterans with a diverse range of lifestyles, ranging from colonial to solitary to parasitoidism. Despite these varied lifestyles, symbioses between these insects and *Streptomyces* have been widely found, and mutualistic insect–bacteria interactions are often described within scientific literature.

**Fig. 1.**
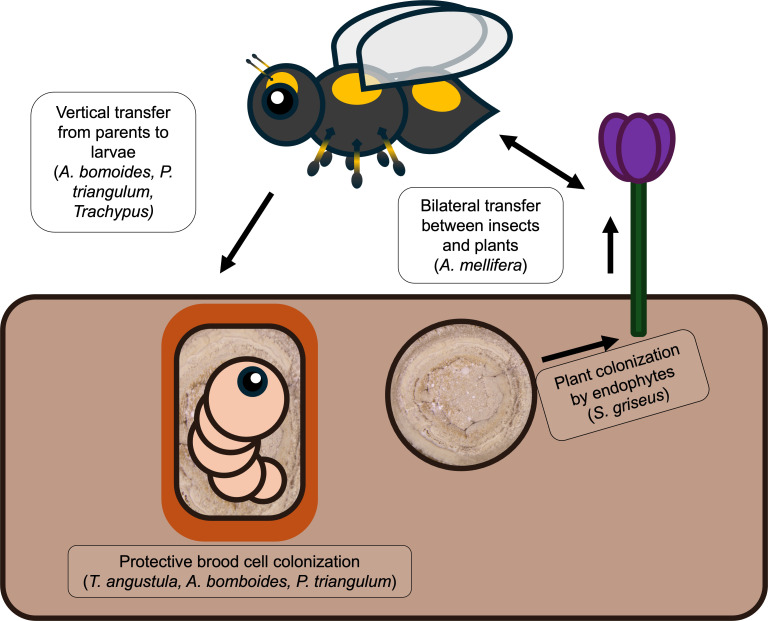
Illustrative summary of interactions between *Streptomyces* and bees/wasps. Arrows indicate directionality of transfer (→ unidirectional colonization; ↔ bidirectional colonization). Colonization of bee and wasp species by *Streptomyces* occurs at all stages of the insect life cycle.

Perhaps the most common perception of bees is as hive-forming, honey-producing, social insects. An example of this lifestyle can be found in *Tetragonisca angustula*, a Meilipona bee native to South America. A study of actinomycetes isolated from the colonies of these bees, as well as from the bees themselves, identified a wide diversity of *Streptomyces* species, as well as other actinomycetes including *Actinomadura* and *Micromonospora* spp. [53]. Interestingly, some of these isolates are closely related to known probiotic *Streptomyces* species isolated from a range of insects. Isolates obtained from *T. angustula* were highly diverse at the taxonomic level, and consequently, the authors attributed the bees as dispersers of these bacteria through their environment. Although not confirmed as *Streptomyces*, hypha-resembling structures have been observed on the surface of *T. angustula* through scanning electron microscopy, suggesting that bees were truly colonized with these strains, rather than transiently carrying spores [53]. Of course, not all bees exist as eusocial insects. *Streptomyces*-bee symbioses have also been demonstrated in solitary bees. An example of this is in the solitary bee species *Anthophora bomboides*, where *Streptomyces* abundance has been demonstrated to increase during winter within the brood cells – underground structures wherein the bee larvae undergo development (Fig. 1). *Streptomyces* spp. isolated from these brood cells demonstrated antifungal bioactivity against multiple known pathogens of * A. bomboides*, implying they have a protective role against fungal infection during larval development [54].

Another example of prophylactic, mutualistic colonization of larvae by *Streptomyces* can be found in the European beewolf (*Philanthus triangulum*). The reproductive strategy of these solitary digger wasps revolves around the capture and paralysis of worker honeybees which their larvae feed on prior to spinning a cocoon for metamorphosis. The beewolf larvae inherit *Streptomyce*s cultivated by antennal secretions from their mother, which they then go on to incorporate within the silk of their cocoon [55]. The presence of these streptomycetes is associated with survival of these larvae into adulthood, through protection of the cocoon from predation by bacteria and fungi which thrive in the warm and humid brood cell environment. The mechanism underpinning this protection has been characterized, with *Streptomyces* colonizing the outside of the young beewolf’s cocoon and producing a cocktail of both antibacterial and antifungal molecules, at concentrations sufficient to inhibit the growth of several pathogenic strains [56]. Antennal colonization by *Streptomyces* is not exclusive to *Philanthus* and has also been observed in other beewolf genera such as *Trachypus* [57] and *Philanthinus* [55]. Members of these three closely related genera are colonized by the same organism: *Streptomyces philanthinus*, suggesting an ancient symbiosis predating their divergence. Genomic analysis of this strain has demonstrated genome erosion consistent with ongoing adaptation to an endosymbiotic lifestyle [58].

Insect-*Streptomyces* symbioses may involve additional parties – a tripartite mutualism has been described between honeybees, strawberry plants and members of the *Streptomyces griseus* group. This fascinating interaction is characterized by mutual inoculations, where endophytic *S. griseus* moves from the root of the strawberry plant to its flower and pollen, where it establishes within the honeybee gut. However, this is not a unidirectional flow of *Streptomyces*; the bees are equally capable of inoculating the flowers that they visit. *Streptomyces* colonization is advantageous for both the bees and the strawberry plants, with colonized strawberry plants benefiting from increased resistance to grey mould infection caused by *Botrytis*, and colonized honeybees are resistant to mortality caused by *Paenibacillus larvae* and *Serratia marcescens* [59]. This highlights the antimicrobial versatility of *Streptomyces* recruitment and interaction with bees.

## *Streptomyces* and *Mammalia*: Chiroptera (bats)

The role of *Streptomyces* in the ecology of more complex organisms is not limited to insects. There is increasing evidence of an ecological role for *Streptomyces* in mammalian ecosystems. One such example is the interaction between *Streptomyces* and bats (Chiroptera). Bats tend to be highly social: forming colonies in caves or on cliffsides for protection. Cave systems are well-known for creating unique environments, usually through the maintenance of high humidity levels and consistent temperature. Microbiome research in bats became a topic of interest with the outbreak of the fungal infection known as white-nose syndrome, caused by *Pseudogymnoascus destructans* (Pd) [60]. The syndrome affected bat colonies throughout eastern North America in the mid-2000s. White-nose syndrome has caused the death of millions of bats, which is of great concern for both bat welfare and their wider role in the ecosystem and agriculture, where they provide natural pest control. Despite the widespread disease caused by *P. destructans*, there is evidence that specific bat colonies in susceptible geographic locations have resisted infiltration from the fungus. Researchers used these colonies to investigate differences between infected and healthy colonies using a combination of 16S rRNA and whole-genome sequencing for species identification, genome mining for potential antibiotic-producing genes, metabolomic studies, and bioassays to confirm activity against *P. destructans*.

*Streptomyces* are common habitants of cave environments and have been observed growing in giant microbial mats on cave surfaces [61]. The high level of microbial activity within caves is not limited to the rock surface. In a recent work, bat wings were examined to determine whether members of the skin microbiome were capable of producing antifungal compounds which may be a contributing factor to the resistance of specific colonies to white-nose syndrome [60, 62–64]. A study by Ham *et al*. [62] isolated 632 bacterial species from various western North American bats (*Antrozous*, *Corynorhinus*, *Eptesicus* and *Myotis* spp.), of these, 422 isolates were identified as *Streptomyces* by 16S rRNA sequencing. Thirty-two *Streptomyces* species were found to be inhibitory against *P. destructans* and 15 novel *Streptomyces* species were identified [62]. Park et al. (2021) further investigated 73 of the isolates from the Hamm *et al*. [62] study [65]. The authors additionally used genome-wide average nucleotide identity (ANI) to further resolve these species, delineating 41 species as a result.

Researchers have been investigating potential probiotics to reduce the mortality of bats caused by *P. destructans*, drawing inspiration from seminal work that showed that *Janthinobacterium lividum* appearing to provide resistance against *Batrachochytrium dendrobatidis* (a fungal pathogen) on salamander skin [66]. Hoyt et al. (2015) also introduced the idea of a probiotic for bats after isolating *Pseudomonas* spp. from bat skin showing complete inhibition of *P. destructans* growth after 40 days at concentrations from 10^6^ to 10^4^ c.f.u. ml^−1^ [60]. A preliminary 16S rRNA bat skin microbiome study comparing white-nose syndrome positive and negative taxa found that genera with known antifungal activity were more abundant within white-nose positive populations, but there was an overall reduction in microbiome diversity [67].

The largest study to date sampled 1,362 bacterial isolates while searching for potential probiotic strains to reduce white-nose syndrome mortality [64]. This study chose 28 isolates as representative known anti-Pd strains and sequenced these using 16S rRNA. *Streptomyces sanglieri*, *S. youssoufiensis*, and a novel *Streptomyces* strain were reported as inhibitors of *P. destructans*.

Genomic studies from Park *et al*. [65] and Montoyo-Giraldo *et al*. (2024) [68] expanded on studies by Hamm *et al*. [62] by applying whole-genome sequencing instead, with Montoyo-Giraldo *et al*., adding a further 59 genomes to the original 73 [65, 68]. The authors reported that the *Streptomyce*s population found within these bats are genetically diverse, with high numbers of biosynthetic gene clusters among those present.

The bat microbiome has been the focus of several studies, with most groups focusing on the characterization of the microbiome using metagenomic sequencing though recently the focus has started to shift towards characterizing the inhibitory activities of members of this bat microbiome. Revisiting a promising strain first isolated by Hamm *et al.* [62] Clements *et al*. [69] completed a metabolomic study using ultra-HPL-MS and antifungal assays to characterize compounds produced by *S. buecherae* [69] and found that nigericin, a known polyether antibiotic (plus chemical analogues), had anti-Pd activity. This study was one of the first metabolomic characterizations of any specific compounds being made by *Streptomyces* alone regarding bat skin and anti-Pd activity. There is potential to combine previous studies’ genome mining for biosynthetic gene clusters to narrow the search for strains with high antifungal potential to carry out further metabolomic studies. The invasion and sustained prevalence of *P. destructans* in North America continues to pose a threat to native bat populations. However, fundamental work highlighting the antifungal activity of *Streptomyces* from bats, and ongoing work to develop a probiotic to reduce mortality caused by these fungal infections, proves to be a promising avenue of exploration. Furthermore, the role of *Streptomyces* in horizontal immunity to fungal diseases of bats exemplifies the chemical ecology which exists between mammals that may be exploited in the future to identify molecules for use in human medicine.

## *Streptomyces* and *Homo sapiens* (humans): an emerging biological interface

Traditionally framed as environmental colonizers and prolific sources of secondary metabolites, *Streptomyces* have largely been studied in the context of soil and microbial ecology. However, this narrow view overlooks their diverse and consequential interactions with humans. Recent sequencing studies suggest that *Streptomyces* are detectable, albeit inconsistently and typically at low abundance, across several human body sites, including the skin, gut and lungs [70, 71]. Given that this genus is rarely recovered by culture from healthy individuals, and metagenomic detection becomes less reliable at low abundance, these findings most likely reflect repeated exposure and transient carriage rather than stable colonization within the human microbiome. Even if transient, their recurring detection across independent datasets suggests that interaction between humans and *Streptomyces* is established.

Within the human gut microbiome, *Streptomyces* are not a dominant genus and are frequently undetected, or only present at very low relative abundance, in 16S rRNA surveys. However, metagenomic studies of the human gut have reported reads assigned to *Streptomyces*, with detection more often described in rural populations and in individuals with closer contact to soils, livestock or agricultural environments than in urban populations [72]. More broadly, industrialized populations tend to show reduced representation of environmentally associated bacteria in the gut microbiome, commonly linked to reduced soil contact, increased food processing and antibiotic exposure [73, 74].

Hunter-gatherer groups may represent an even stronger example of this, with routine interaction with natural environments and fewer of the industrial factors that shape gut microbiome composition. In metagenomic data from the Hadza hunter-gatherers, Otani et al. (2025) reported a high fraction of reads mapping to *Streptomyces* compared with westernized populations, consistent with greater environmental exposure rather than stable colonization [75].

Environmental exposure is the most likely route by which *Streptomyces* enter the human gut. In practice, this likely occurs via ingestion of soil-associated material on food and consumption of untreated water, both of which create repeated opportunities for introduction [76]. Several studies have discussed soil-to-gut transmission as a contributor to microbiome composition in rural settings, but for spore-forming organisms such as *Streptomyces* it is often difficult to separate true colonization from simple passage through the gastrointestinal tract. The pattern seen across datasets of intermittent detection and low relative abundance fits better with transient passage following recent exposure than with stable colonization [76].

Evidence from ancient microbiome studies provides insights into historical human exposure to *Streptomyces*. Ancient DNA isolated from coprolites and dental calculus has revealed environmental micro-organisms that are rare in modern industrialized microbiomes. While *Streptomyces*-specific identifications are uncommon, likely due to limitations in resolution and reference databases, related *Actinomycetota* have been identified [77, 78]. Nonetheless, the frequency with which soil-associated bacteria appear in ancient microbiomes is consistent with greater exposure to soil-associated micro-organisms, including *Actinomycetota*, in earlier contexts [79, 80].

Historical evidence also supports repeated human exposure to antibiotics produced by environmental *Streptomyces*. In skeletal remains of a population in Sudanese Nubia (c. 350–550 CE), Bassett *et al*. reported fluorescence banding patterns consistent with tetracycline incorporation into bone, which was interpreted as dietary exposure rather than post-mortem contamination [81]. Subsequent work using liquid chromatography-MS recovered tetracycline from Nubian skeletons, supporting ante-mortem exposure to these compounds [82]. A proposed route of exposure is ingestion via cereal grains used for beer production, providing a possible mechanism by which tetracycline-producing soil-associated *Streptomyces* could repeatedly enter the human microbiome. These findings are consistent with long-term, environmentally mediated contact between humans and antibiotic-producing *Actinomycetota* and highlight that historical interactions may be detectable through exposure to secondary metabolites even where *Streptomyces* DNA is not recoverable.

Studies relating to *Streptomyces* in the human gut microbiome are limited by technical challenges related to the genus. Additionally, many metagenomic datasets are often skewed toward urban populations in the global north, limiting inference about traditional-living populations where environmental exposure is higher, and *Streptomyces* detection may be more likely [73].

*Streptomyces* are relevant to human gut microbiome studies because of their specialized metabolism and the range of bioactive compounds they produce. Beyond their well-documented antibiotic production, they are capable of synthesizing a large range of specialized metabolites including immunosuppressives and anti-cancer compounds [83, 84]. The idea of *Streptomyces* producing compounds that may be useful to humans coincides with the ‘old friends’ hypothesis, in which reduced exposure to immunoregulatory environmental microbes in industrialized settings contributes to immune dysregulation. However, functional roles of *Streptomyces* are yet to be elucidated, with limited direct evidence for *in vivo* activity.

In summary, the evidence suggests that *Streptomyces* are detectable but rare members of the human gut microbiome, with increased prevalence in agricultural and ancient populations. The genus’ presence is best explained by transient environmental exposure rather than stable colonization. While their capacity for secondary metabolite production raises questions about their potential roles in host–microbe interactions, current data do not support a central functional role in the human microbiome; however, as metagenomic sequencing technologies improve resolution and detection, a role for *Streptomyces* may become clear.

## *Streptomyces* as human pathogens

As previously mentioned, there is accumulating evidence that suggests *Streptomyces* species can be detected in animals, including humans, across a range of anatomical sites. Reports have described the recovery *of Streptomyces* from the human gastrointestinal tract, respiratory tract and skin [85, 86]. Clinical investigations involving infection of immunocompromised hosts are more likely to establish *Streptomyces* as the causative agent of disease, rather than from systematic surveys of healthy individuals. Large-scale microbiome studies of healthy populations rarely identify *Streptomyces* as a consistent or prevalent member of the normal microbiota, suggesting that its detection is more commonly associated with disease states or opportunistic/transient colonization rather than stable commensalism. This contrasts with other *Actinomycetota* taxa, such as *Cutibacterium* spp., which are well established as components of the normal skin microbiota. Methodological limitations may further contribute to the underrepresentation of *Streptomyces* in healthy-host studies, as their filamentous morphology, slow growth and relatively low abundance may reduce detection using standard culture-based or short-read sequencing approaches.

Despite their primarily saprophytic lifestyle, *Streptomyces* species possess a documented, albeit rare, capacity to cause disease in humans and animals. Taxonomically, they are related to several clinically important genera such as *Nocardia*, *Actinomyce*s and *Dermatophilus* [87, 88]. Members of this order are associated with actinomycotic mycetoma, a chronic and progressively destructive infection of subcutaneous tissues that typically arises following traumatic inoculation of environmental bacteria through breaches in the skin barrier [89]. Clinically, mycetoma is characterized by localized swelling, sinus tract formation and the discharge of granules containing filamentous bacteria. Lesions most commonly affect the lower extremities, although involvement of the hands, face and neck is also reported [90].

In humans, actinomycotic mycetoma caused by *Streptomyces* species is most frequently documented in tropical and subtropical regions, particularly in Sub-Saharan Africa. *Streptomyces somaliensis* and *Streptomyces sudanensis* are recognized as major etiological agents in these endemic regions [88]. Human infections are often associated with immunocompromised states, reinforcing the opportunistic nature of *Streptomyces* pathogenicity.

Although cutaneous disease predominates, invasive infections have been reported, including sacroiliitis caused by *S. griseus* [85], peritonitis associated with *Streptomyces viridis* [91] and a well-documented case of invasive *Streptomyces* infection was reported by Rose *et al*. following a penetrating skull injury complicated by fracture, haematoma and pneumocephalus [92]. This case underscores the capacity of *Streptomyces* to cause severe central nervous system infection following traumatic inoculation, further highlighting the clinical significance of *Streptomyces* outside classical mycetoma presentations.

Reports of *Streptomyces-*associated disease in animals are comparatively scarce, but are likely underrepresented due to low clinical suspicion and diagnostic challenges. Confirmed veterinary cases have been described in cats, dogs, donkeys, dolphins and horses [87–93], frequently involving atypical clinical presentations and prolonged disease courses. Notably, several cases have identified novel or unexpected *Streptomyces* species as causative agents. These include pyogranulomatous dermatitis in a dog associated with *Streptomyces cyaneus*^87^ and fistulous infections in donkeys caused by multiple *Streptomyces* species [93]. The identification of novel species in these cases suggests that the pathogenic potential of *Streptomyces* in animals may be broader than currently appreciated.

A major challenge in recognizing *Streptomyce*s-associated infections is their frequent dismissal as environmental contaminants when isolated from clinical specimens. Given their ubiquity in soil and air, *Streptomyce*s isolates are often overlooked during diagnostic workups, leading to delayed or inappropriate treatment. The increasing application of molecular diagnostic techniques, including 16S rRNA gene sequencing and whole-genome analysis, has improved species-level identification and is likely to reveal additional cases previously unrecognized [94].

## Understanding *Streptomyces* virulence in human disease

The mechanisms underlying *Streptomyces* pathogenicity remain poorly defined. Unlike traditional pathogens, *Streptomyces* species lack clearly defined virulence factors and are thought to only cause disease opportunistically. Recent genomic analyses have identified mammalian cell entry (*mce*) gene homologues in several *Streptomyces* species. These genes were first identified in *Mycobacterium tuberculosis* (Mtb) and are broadly conserved across both pathogenic and non-pathogenic members of the *Actinomycetales* order (Casali *et al*., 2007). Mtb encodes four distinct *mce* loci, whereas *Streptomyces* species possess a single *mce* gene cluster that shares homology with components of the four Mtb *mce* operons (Clark *et al*., 2013). A single copy of this cluster has been identified in *S. avermitilis* MA-4680, *Streptomyces coelicolor* A3(2)/M145 [95–97] and *Streptomyces ambofaciens* ATCC 23877 (2015).^98^

**Fig. 2.**
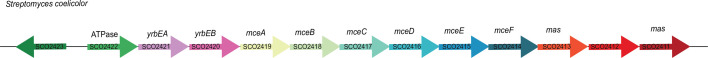
Schematic diagram of mce operon from *S. coelicolor*.

In *S. coelicolor*, the mammalian cell entry gene cluster is 13.4 kb long and comprises eight conserved core genes (*mceABCDEF, yrbEA* and *yrbEB*), two conserved *mce-*associated genes (*mas*) and one gene encoding a domain of unknown function (Fig. 2) [96]. This organization resembles the Mtb *mce1* operon, which likewise contains two *yrbE* genes and eight core *mce* genes. In *Mycobacterium smegmatis*, the Mce1 system assembles into an elongated complex spanning from the mycobacterial outer membrane to the inner membrane and extending ~225 Å into the periplasmic space. The channel formed by the complex is lined with hydrophobic residues that facilitate lipid transport [99]. *Streptomyces mce* genes are predicted, based on homology, to encode a comparative transmembrane structure [97], although no structure has yet been experimentally resolved. Consistent with a lipid transport role, thin-layer chromatography experiments show Δ*mce1* mutants in Mtb (H37Rv) accumulate excess mycolic acids and display an altered lipid profile [100]. Furthermore, co-culture of *Streptomyces* with mycolic acid-producing bacteria alters the secondary metabolite profile, redirecting metabolism towards natural product biosynthesis [101]. These interactions may support *Streptomyces* survival within the soil niche or within the rhizosphere, which can be colonized with mycolic acid-producing *Mycobacterium* [102]. Together, these observations suggest that *mce* genes may contribute to environmental persistence in *Streptomyces*.

Disruption of the genes within the *mce1* operon in Mtb prevents the establishment of persistent infection, and multiple studies have shown that specific Mce proteins enhance the ability of Mtb to enter mammalian cells. The original discovery of *mce1* in Mtb demonstrated that expression of *mce*1A in non-pathogenic *E. coli* increased invasion of non-phagocytic HeLa cells [103] and later work showed enhanced uptake of polystyrene latex microspheres [104]. Additional studies established that Δ*mce1* mutants exhibit hyper-replicative phenotypes and fail to transition into a chronic infection state [105], further supporting the link between *mce* genes and virulence. In contrast, there is limited evidence that *Streptomyces mce* genes influence host-cell attachment and invasion. One study co-culturing *S. coelicolor* Δ*mce1* mutants with *Acanthamoeba polyphaga,* showed that loss of the *mce* gene cluster resulted in rapid spore germination within the acidic vacuole, resulting in amoeba death within 24 h [96]. This study supports that, similar to *Mycobacterium*, *Streptomyces mce* mutants may also experience hyper-replicative phenotypes. Despite their infrequent association with disease, *Streptomyces* species have been implicated in several aforementioned, opportunistic infections underscoring the importance of assessing whether *mce* genes contribute to virulence in these cases.

The widespread presence of *mce* genes across *Actinomycetales*, combined with phylogenetic analyses indicate that all eight identified *mce* gene clusters derive from duplication of an ancestral operon which originally contained six genes [97]. Given that *Streptomyces* are estimated to have split from the common ancestor over 440 million years ago, the operon’s origins are likely ancient, with subsequent speciation and gene duplication events shaping its divergence in both function and expression patterns [96]. Understanding the evolutionary trajectory and diversity of the *mce* operon in *Mycobacterium* and other *Actinomycetales* may inform on the mechanisms that enable opportunistic infection by *Streptomyces* species.

## Conclusion and outlook

Overall, interactions between *Streptomyces* and the animal kingdom represent an underexplored interface between environmental microbiology and infectious disease. Relationships between *Streptomyces* and insects are comparatively well characterized, with both the taxonomy and chemical ecology of these associations well resolved. In contrast, major gaps remain in our understanding of the roles of *Streptomyces* within mammalian microbiomes, as well as their pathogenic potential and contributions to disease. From an evolutionary perspective, it is plausible that *Streptomyces* could occupy functional niches within the human microbiome, given their established associations with other mammals and diverse ecosystems, and their capacity to produce natural products that are often highly specialized for interactions with human molecular targets. One area warranting further study is whether *Streptomyces* derive benefits from animal associations, and if so, what those benefits may be. Proposed hypotheses include animal-mediated dispersal, which may be advantageous given the lack of traditional bacterial motility in *Streptomyces*, as well as access to stable, nutrient-rich niches within animal microbiomes that support growth and persistence. Improved diagnostic awareness, together with targeted investigations of host-microbe interactions and systematic surveys of healthy animal populations, will be essential to truly explore the ecological role of *Streptomyces* across the animal kingdom [106].

## Abbreviations

BN: bacterivorous nematode
mce: Mammalian cell entry genes
Mtb: *Mycobacterium tuberculosis*
Pd: *Pseudogymnoascus destructans*
PNN: plant-parasitic nematode
